# Trimethylsilanol Cleaves Stable Azaylides As Revealed by Unfolding of Robust “Staudinger” Single-Chain Nanoparticles

**DOI:** 10.1021/acspolymersau.3c00046

**Published:** 2024-01-09

**Authors:** Agustín Blázquez-Martín, Sebastián Bonardd, Ester Verde-Sesto, Arantxa Arbe, José A. Pomposo

**Affiliations:** † Centro de Física de Materiales (CSIC - UPV/EHU) − Materials Physics Center MPC, P° Manuel Lardizabal 5, E-20018 Donostia, Spain; ‡ Departamento de Polímeros y Materiales Avanzados: Física, Química y Tecnología,University of the Basque Country (UPV/EHU), P° Manuel Lardizabal 3, E-20800 Donostia, Spain; § IKERBASQUE − Basque Foundation for Science, Plaza Euskadi 5, E-48009 Bilbao, Spain

**Keywords:** azaylides, Staudinger reaction, unfolding, single-chain nanoparticles, SCNPs

## Abstract

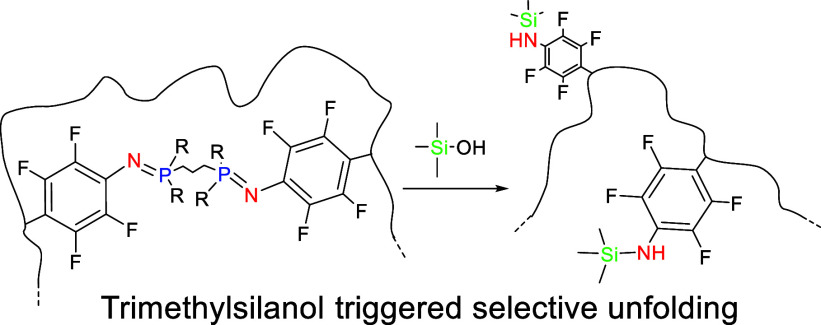

Herein, we disclose a unique and selective reagent for the cleavage of stable azaylides prepared by the nonhydrolysis Staudinger reaction, enabling the on-demand unfolding of robust single-chain nanoparticles (SCNPs). SCNPs with promising use in catalysis, nanomedicine, and sensing are obtained through intrachain folding of discrete synthetic polymer chains. The unfolding of SCNPs involving reversible interactions triggered by a variety of external stimuli (e.g., pH, temperature, light, and redox potential) or substances (e.g., competitive reagents, solvents, and anions) is well known. Conversely, methods for the unfolding (i.e., intrachain disassembly) of SCNPs with stronger covalent interactions are scarce. We show that trimethylsilanol (Me_3_SiOH) triggers the efficient unfolding of robust “Staudinger” SCNPs with stable azaylide (−N=P−) moieties as intrachain cross-linking units showing exceptional stability toward water, air, and CS_2_, a standard reagent for azaylides. As a consequence, Me_3_SiOH arises as a rare, exceptional, and valuable reagent for the cleavage of stable azaylides prepared by the nonhydrolysis Staudinger reaction.

## Introduction

Intrachain folding of isolated, discrete synthetic polymer chains gives to single-chain nanoparticles (SCNPs).^1−7^ SCNPs are promising nano-objects for the development of new enzyme-mimetic catalysts, improved sensing nanomaterials, innovative drug delivery vehicles, and so on.^8^ For instance, Barner-Kowollik and co-workers^9^ reported SCNPs capable of catalyzing the photo-oxidation of nonpolar alkenes under green light up to three times more efficiently than an equivalent small-molecule photosensitizer at an identical concentration. Binder and co-workers^10^ developed near-infrared (NIR)-fluorescent SCNPs that change their photophysical behavior upon heating and generate a fluctuating photoacoustic (PA) signal which can be used as a novel effect for PA imaging. Moreover, Paulusse and co-workers^11^ showed rapid uptake by HeLa cancer cells of SCNPs via polyplex formation and cytosolic delivery of doxorubicin from the SCNPs acting as nanocarriers. For recent reviews about the full range of possibilities SCNPs offer in different applications, we refer the reader to previous studies.^12−21^

When the intrachain folding process in a synthetic polymer chain is induced through reversible interactions, unfolding of the resulting SCNPs (i.e., intrachain disassembly) is possible under certain conditions.^22^ For instance, SCNPs folded through hydrogen bonding (HB) interactions were found to unfold upon acidification,^23^ by addition of a competitive HB compound,^24−26^ anion,^27^ solvent,^28^ or by increasing temperature.^28,29^ Additionally, reversible SCNPs have been unfolded triggered by addition of a base,^30^ under interrupted illumination (i.e., dark conditions),^31^ by losing CO_2_ molecules^32,33^ or upon external voltage stimuli.^34^ Complementary, SCNPs folded through dynamic covalent bonds like disulfide^35,36^ or enamine bonds^37^ went back to their linear precursor chains in the presence of reducing agents or by lowering the pH, respectively. The decrease in activity of some catalytic SCNPs in vivo was attributed to the spontaneous unfolding of these reversible SCNPs in these complex media.^38^

Conversely, robust SCNPs result when the intrachain folding process involves the generation of covalent interactions.^39^ Unfolding (intrachain disassembly) of robust SCNPs could be a useful method to modulate SCNP activity/functionality. However, only a few examples of the unfolding of robust SCNPs have been reported. In pioneering work, Temel and co-workers^40^ synthesized SCNPs through intrachain cross-linking with Michler’s ketone units via Menschutkin chemistry that unfold to the linear precursor upon photoirradiation at a wavelength of λ = 365 nm. Barner-Kowollik and co-workers^41^ reported the unfolding triggered by *meta*-chloroperbenzoic acid (mCPBA) of fluorescent SCNPs photo-cross-linked at λ = 320 nm. The same group disclosed the chemiluminescent self-reported unfolding of peroxyoxalate-containing SCNPs triggered by hydrogen peroxide^42^ and, more recently, SCNPs containing fluorescent bimane moieties as cross-linking units that can be fully unfolded using visible light (λ = 415 nm).^43^ Previously, Studer and co-workers^44^ demonstrated λ-orthogonal folding (at λ = 415 nm) and unfolding (at λ = 254 nm) of SCNPs based on the photoclick reaction of acylsilanes with indoles. Critically, the development of new methods for unfolding robust SCNPs is still a challenging task. In this work, we report the unfolding triggered *selectively* by trimethylsilanol (Me_3_SiOH) of robust “Staudinger” SCNPs with stable azaylide (−N=P−) moieties as intrachain cross-linking units.

**Scheme 1:**
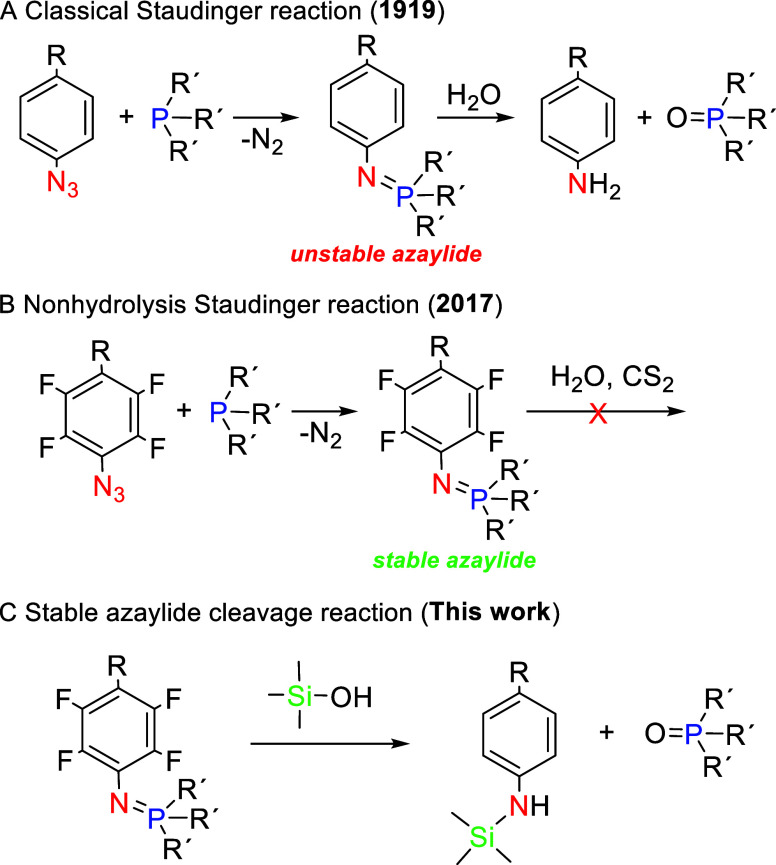
(A) Staudinger Reaction; (B) Nonhydrolysis Staudinger Reaction Leading to a Robust Azaylide; (C) Stable Azaylide Cleavage Reaction (This Work)

Phosphorus (P) chemistry, with broad academic and industrial significance, is useful in several fields from catalysis and polymer science to pharmaceutical applications. In particular, the Staudinger reaction^45^ is the coupling of an azide with a tertiary phosphine to give, after N_2_ release, an iminophosphorane (i.e., azaylide) that, in the presence of water, hydrolyzes to a primary amine and phosphine oxide (Scheme 1A). This reaction that proceeds in nearly full conversion without the need for any catalyst has found broad application in organic synthesis and, as pioneered by Bertozzi and co-workers,^46^ also in chemical biology.

**Scheme 2:**
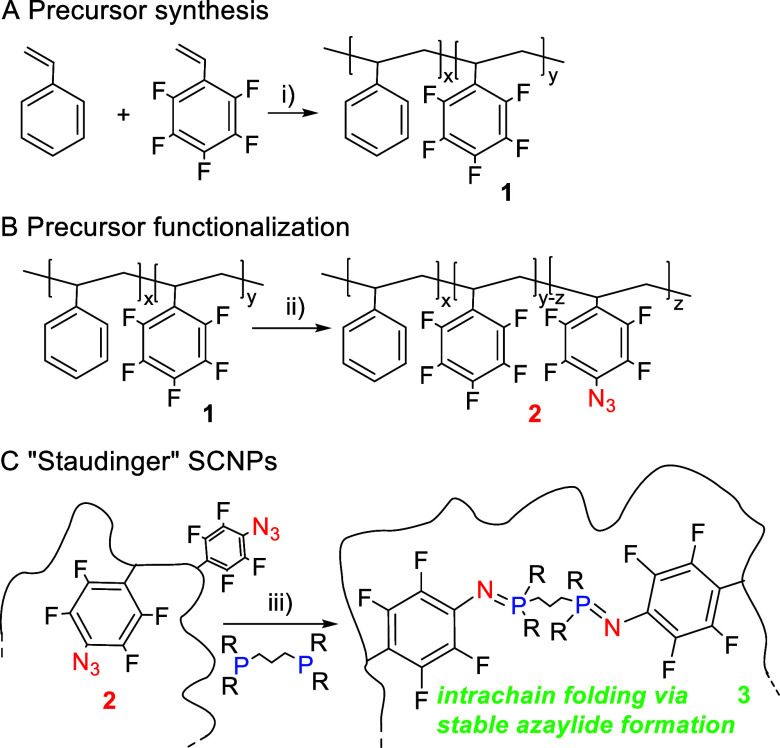
(A) Synthesis of a Styrenic Copolymer Containing Pentafluorophenyl Moieties, **1**, as the Initial Precursor of “Staudinger” SCNPs; (B) Partial Azidation of Pentafluorophenyl Moieties to *p*-Azido-tetrafluorophenyl Pendants To Obtain the Functionalized Precursor, **2**; (C) Intrachain Folding of **2** to Robust “Staudinger” SCNPs, **3**, with Stable Azaylide (−N=P−) Moieties as Intrachain Cross-Linking Units Reaction conditions: (i) bulk, AIBN, CMDTC, 65 °C, 15 h; (ii) NaN_3_, DMF, 80 °C, 24 h; (iii) THF, r.t., 24 h.

Recently, several strategies to stabilize the azaylide intermediate of the Staudinger reaction against hydrolysis, oxidation, and aza-Wittig reactions have been reported. Hence, Ramström, Yan and co-workers^47^ discovered that the use of perfluoroaryl azides in the Staudinger reaction leads to the rapid formation of stable iminophosphoranes in high yield. Similarly, Yi and co-workers reported a fast nonhydrolysis Staudinger reaction based on the use of *o,o*′-difluorinated aromatic azides^48^ that becomes a very fast reaction when tetrafluorinated aromatic azides are employed.^49^ Additionally, the formation of robust, water- and air-stable azaylides through the Staudinger reaction between electron-deficient aromatic azides such as *o*,*o*′-dichlorophenyl azide and triarylphosphines was reported by Yoshida, Hosoya and co-workers.^50^ In the case of organic compounds with *p*-azido-tetrafluorophenyl moieties, the azaylides investigated were so stable over time that they did not hydrolyze in the presence of water and did not react when mixed for 35 days with an excess of CS_2_, a standard reagent for azaylides (Scheme 1B).^47^ Moreover, the synthesis of polyphosphazenes with high thermal stability by the fast nonhydrolysis Staudinger reaction between a bis-perfluoroaryl azide and a bis-phosphine was additionally reported by Yan and co-workers.^51^ More recently, Yamashina, Toyota and co-workers^52^ used the nonhydrolysis Staudinger reaction for the synthesis of robust azaylide-based amphiphiles that are stable against hydrolysis in water and form ca. 2 nm spherical aggregates through self-assembly. To the best of our knowledge, no specific reagent to cleave stable azaylides prepared by the nonhydrolysis Staudinger reaction has been reported yet. Herein, we disclose that Me_3_SiOH is a rare, exceptional, and valuable reagent for the cleavage of stable azaylides prepared by the nonhydrolysis Staudinger reaction, as revealed by the efficient unfolding of robust “Staudinger” SCNPs containing stable azaylide (−N=P−) moieties as intrachain cross-linking units prepared according to Scheme 2.

## Results and Discussion

**Figure 1:**
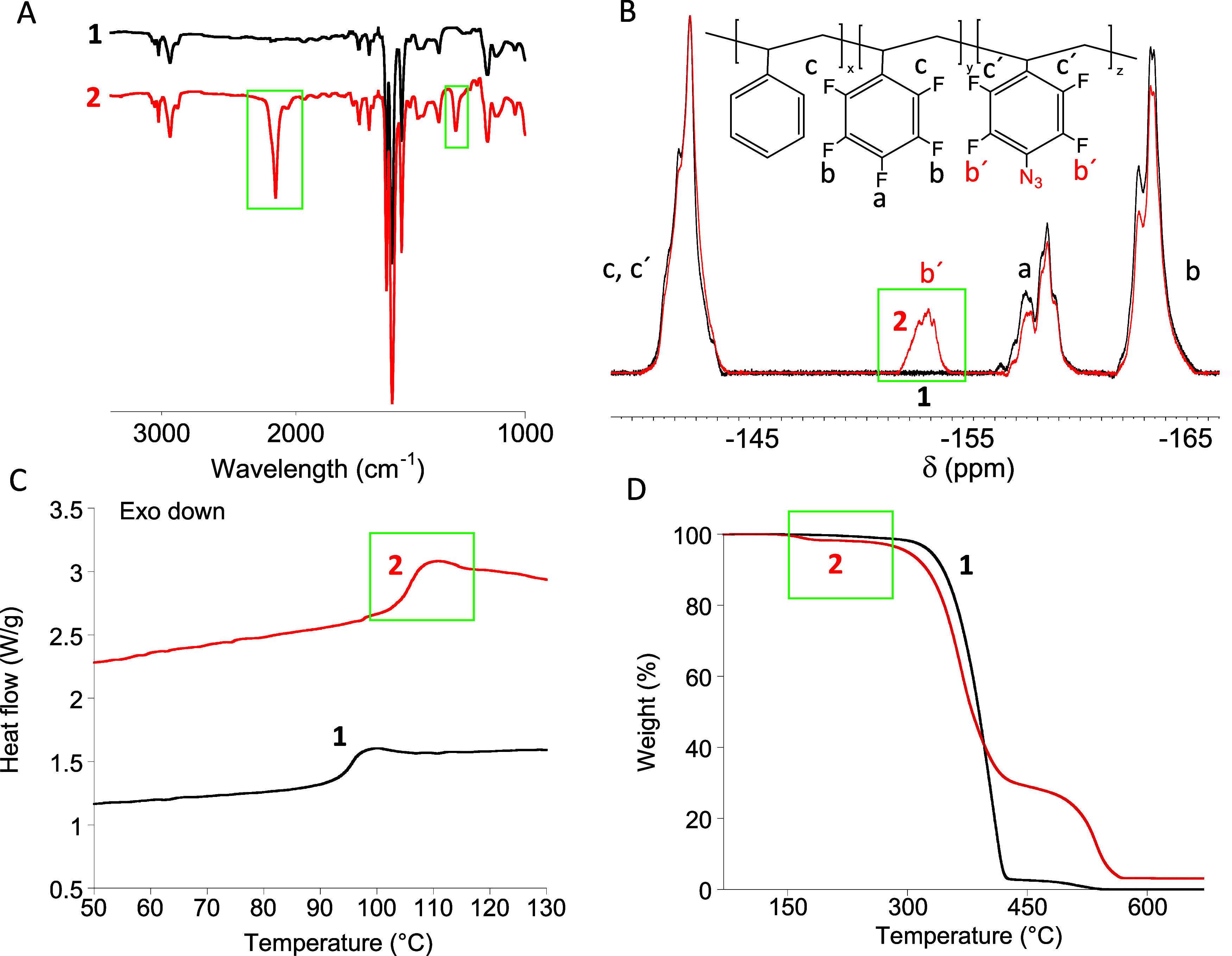
(A) Infrared (IR) spectra of the initial precursor, **1**, and the functionalized precursor of “Staudinger” SCNPs, **2**. (B) ^**19**^F nuclear magnetic resonance (NMR) spectra of **1** and **2**. (C) Differential scanning calorimetry (DSC) traces of **1** and **2**. (D) Thermogravimetric analysis (TGA) traces of **1** and **2**.

As an initial precursor of “Staudinger” SCNPs, we synthesized a styrenic copolymer, **1**, through radical addition–fragmentation chain-transfer (RAFT) polymerization of styrene (S) and pentafluorostyrene (PFS) (see Scheme 2A and Experimental Section). Since the monomer reactivity ratios of S and PFS are reported^53^ to be *r*_S_ = 0.43 and *r*_PFS_ = 0.22, the copolymer is expected to show moderate alternating behavior as a consequence of the relatively low value of *r*_S_ × *r*_PFS_ = 0.095. **1**—that was obtained in 61% yield—showed a PFS molar content of 32 mol % as determined by elemental analysis (EA), a weight-average molecular weight of *M*_w_ (multiangle laser light scattering (MALLS) detector) = 42.6 kDa, and a low dispersity of *Đ* = 1.08 (see SI). Initial precursor **1** was functionalized through the azide–*para*-fluoro substitution reaction (see Scheme 2B). This reaction, originally developed for photoaffinity labeling by Keana and Cai,^54^ was recently implemented for efficient sequential postpolymerization modification by Roth and co-workers.^55^ Through azide–*para*-fluoro substitution, the functionalized precursor **2** was obtained in very good yield (91.6%), with a content of *p*-azido-tetrafluorophenyl units of 11 mol % as determined by EA, *M*_w_ (MALLS) = 50.9 kDa, and *Đ* = 1.13 (see Supporting Information, SI). As illustrated in Figure 1A, the infrared (IR) spectrum of **2** shows the appearance of two new bands when compared to the IR spectrum of **1**: an intense band centered at ca. 2135 cm^–1^ corresponding to the asymmetric N=N=N stretching vibration of the azide functional group, and a less intense band at ca. 1225 cm^–1^ from the symmetric stretching vibration of this group. Complementary, a new band centered at −152.9 ppm (denoted as **b′** in Figure 1B) is observed in the ^19^F nuclear magnetic resonance (NMR) spectra of **2** arising from the two fluorine atoms near the azide moiety in each *p*-azido-tetrafluorophenyl unit. Integration of the areas corresponding to peaks **b′** (*A*_b′_) and **b** (*A*_b_) in Figure 1B allows one to estimate the molar content of *p*-azido-tetrafluorophenyl units as [*A*_b′_/(*A*_b′_ + *A*_b_)] × 100. The azidation degree estimated from ^19^F NMR was in very good agreement with that obtained from the EA data. The azide–*para*-fluoro substitution reaction was found to have a profound effect on the thermal behavior of the functionalized precursor **2**, as illustrated in Figure 1C,D. On one hand, partial azidation of the initial precursor increases the glass transition temperature (*T*_g_) from 95 (**1**) to 105 °C (**2**) probably due to dipolar interactions between the residual pentafluophenyl and the new *p*-azido-tetrafluorophenyl moieties reducing the segmental mobility of **2**. On the other hand, azidation of **1** leads to a significant decrease in thermal stability as observed in Figure 1D. The functionalized precursor **2** is thermally stable up to ca. 150 °C so above this temperature loss of dinitrogen takes place due to the thermally labile nature of the *p*-azido-tetrafluorophenyl moieties.^54^ All the above results support the successful preparation of an appropriate styrenic precursor of “Staudinger” SCNPs with 11 mol % of *p*-azido-tetrafluorophenyl pendants. It is worth mentioning that SCNPs are typically synthesized from precursors containing between 10 and 30 mol % of functional groups.

**Figure 2:**
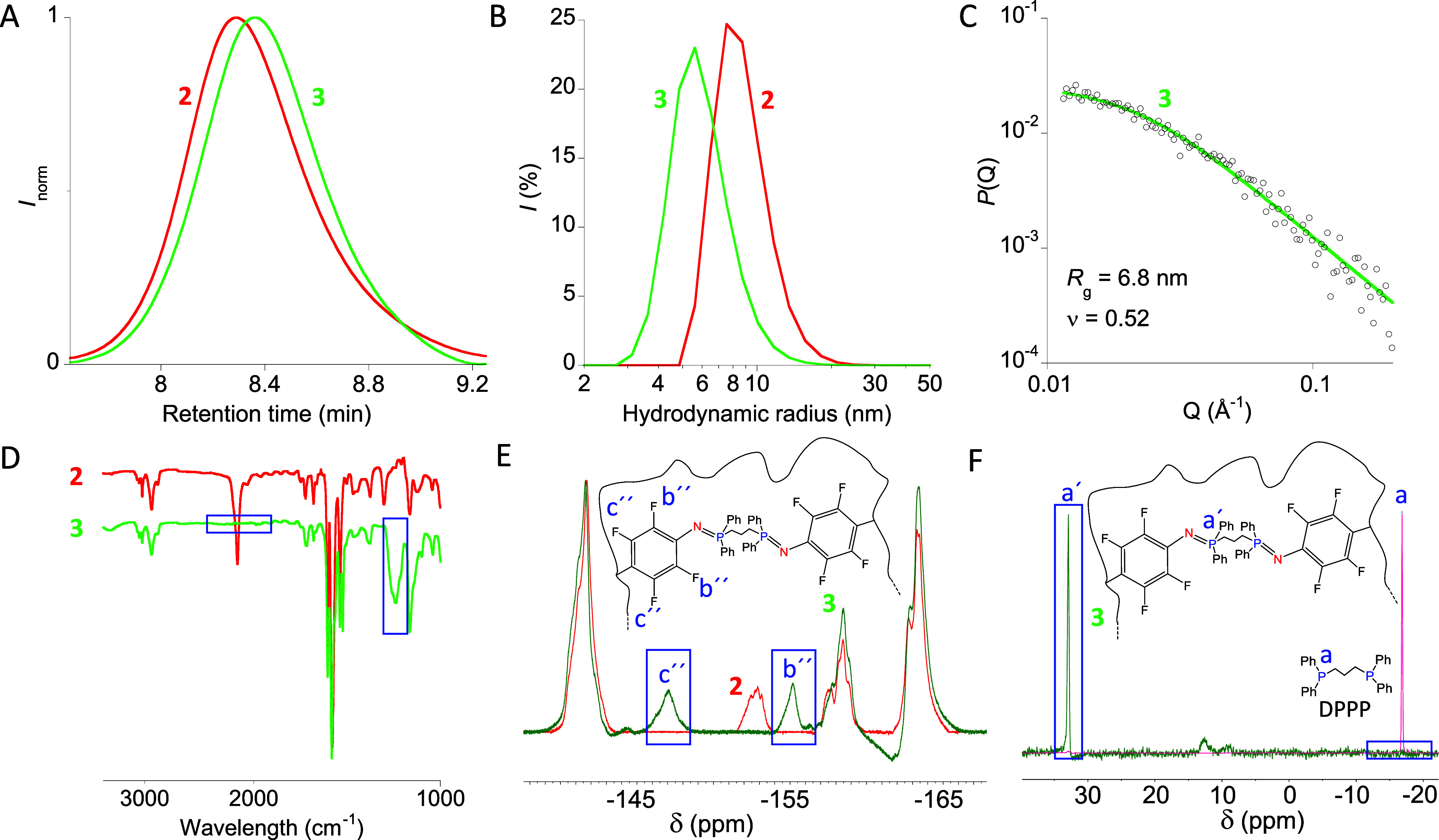
(A) Size exclusion chromatography (SEC) traces (differential refractive index (DRI) detector, THF, 1 mL min^–**1**^) of the functionalized precursor **2** and “Staudinger” SCNPs, **3**, synthesized from **2** via stable azaylide intrachain formation with 1,3-bis(diphenylphosphino)propane (DPPP) as bifunctional cross-linker. (B) Dynamic light scattering (DLS) size distributions of **2** and **3**. (C) Small-angle X-ray scattering (SAXS) results of “Staudinger” SCNPs **3**. (D) IR spectra of **2** and **3**, showing the latter the complete disappearance of the asymmetric N=N=N stretching vibration band centered at 2135 cm^–**1**^ and the appearance of the N=P stretching vibration band centered at 1175 cm^–**1**^. (E) ^**19**^F NMR spectra of **2** and “Staudinger” SCNPs, **3**, with new bands coming from −F atoms at the *orto*- and *meta*-position with respect to azaylide groups denoted as **b″** and **c″**, respectively. (F) ^**31**^P NMR spectra of DPPP (δ = −16.9 ppm) and “Staudinger” SCNPs **3** (δ = 32.9 ppm). The weak signal at ∼12 ppm can be attributed to a phosphine oxide impurity.

For the folding of **2** to robust “Staudinger” SCNPs, **3**, via stable azaylide intrachain formation we selected 1,3-bis(diphenylphosphino)propane (DPPP) as a bifunctional cross-linker (see Scheme 2C). The synthesis was carried out at room temperature for 24 h in degassed THF by means of the efficient continuous addition technique (addition rate: 30 mL h^–1^) (see Experimental Section for details). We isolated the “Staudinger” SCNPs, **3**, by precipitation in cold hexane and further drying under vacuum at 35 °C (**3**: yield = 94.4%, *M*_w_ (MALLS) = 61.4 kDa, *Đ* = 1.04). As illustrated in Figure 2A–C, all results from size exclusion chromatography (SEC), dynamic light scattering (DLS), and small-angle X-ray scattering (SAXS) supported the successful single-chain nanoparticle formation. A shift toward longer retention time and, hence, smaller hydrodynamic radius (*R*_h_) was observed by SEC (see Figure 2A).^8^ DLS measurements confirmed a reduction in the average hydrodynamic size from *R*_h_(**2**) = 9.4 nm to *R*_h_(**3**) = 6.3 nm. Complementary, SAXS measurements provided values of the radius of gyration (*R*_g_ (**3**) = 6.8 nm) and size-scaling exponent (ν (**3**) = 0.52 vs ν (**2**) ≈ 0.6) that supported the efficient folding of the functionalized precursor **2** to SCNPs **3**. Confirmation of the quantitative consumption of *p*-azido-tetrafluorophenyl units in **2** to give stable azaylide-based cross-linking points in **3**, according to Scheme 2C, was obtained from IR, ^19^F, and ^31^P NMR spectroscopy (cross-linking degree of SCNPs **3**: 11 mol %). Figure 2D illustrates the complete disappearance of the asymmetric N=N=N stretching vibration band at 2135 cm^–1^ upon formation of the “Staudinger” SCNPs, and the appearance of a new band in the IR spectrum of **3** centered at 1175 cm^–1^ corresponding to the N=P stretching vibration. Similarly, the ^19^F NMR band at δ = −152.9 ppm corresponding to two fluorine atoms near the azide moiety in each *p*-azido-tetrafluorophenyl unit of **2** was found to completely disappear upon the formation of SCNPs **3** (Figure 2E). Instead, the ^19^F NMR spectrum of **3** showed two new peaks centered at δ = −155.2 and −147.1 ppm arising from −F atoms at the *orto*- and *meta*-positions with respect to azaylide groups, respectively. Moreover, the ^31^P NMR spectrum of **3** revealed an intense peak centered at δ = 32.9 ppm arising from the N=P cross-linking points. No sign of unreacted bisphosphine (δ = −16.9 ppm) was found in the ^31^P NMR spectrum of the “Staudinger” SCNPs (Figure 2F). Taken together, the above results confirm the successful folding of functionalized precursor **2** to “Staudinger” SCNPs **3** via intrachain azaylide formation. Additional characterization data of **3** are included in the SI.

**Figure 3:**
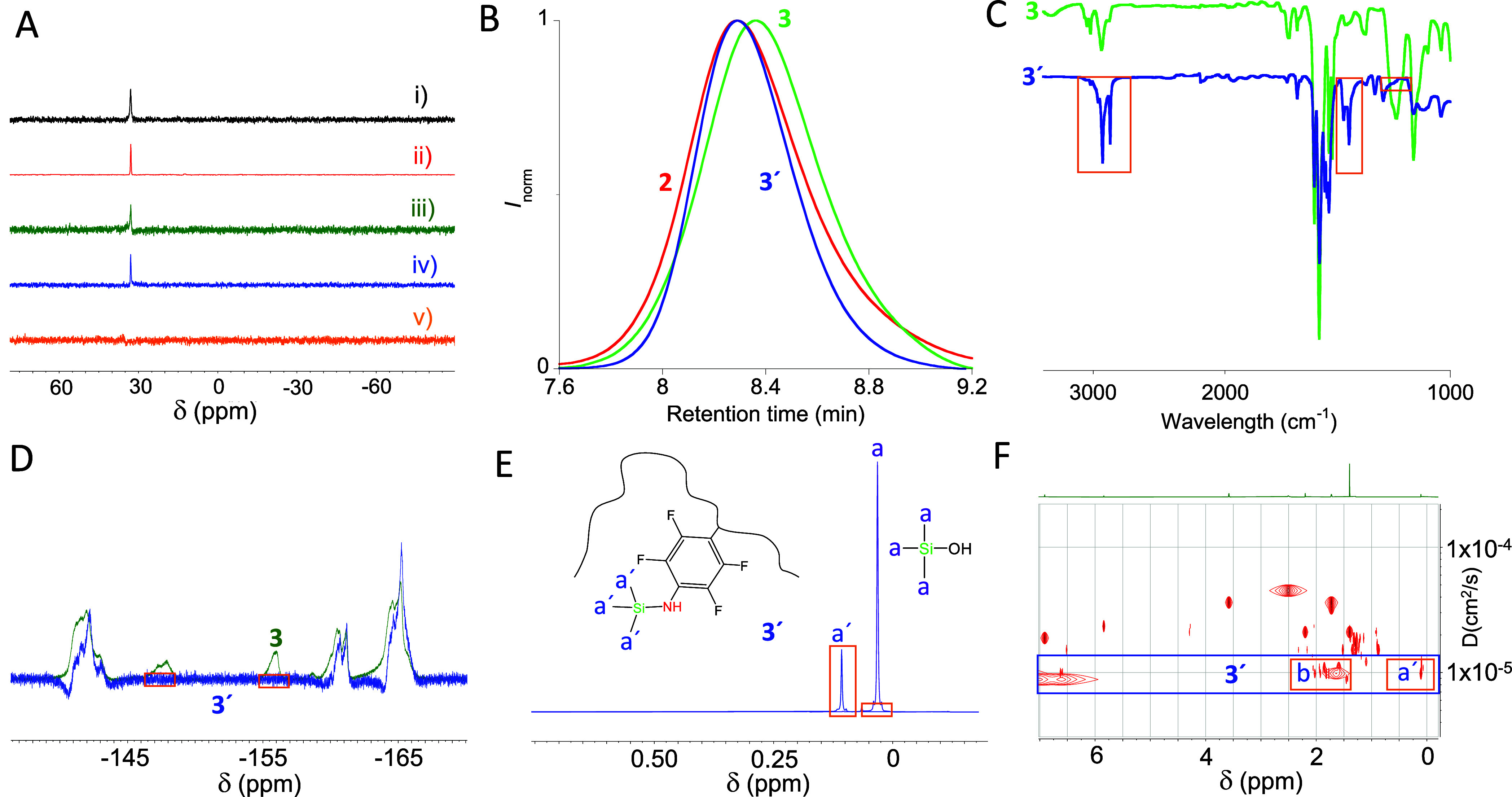
(A) ^**31**^P NMR spectra of robust “Staudinger” SCNPs after: (i) conventional thermal heating at 120 °C for 3 days in DMF, (ii) microwave heating at 120 °C for 1 h in DMF, (iii) 3 days in THF at r.t. in the presence of an excess of CF_3_COOH, (iv) 3 days in THF at r.t. in the presence of an excess of CS_2_, (v) 3 days in THF at r.t. in the presence of an excess of Me_3_SiOH. (B) Comparison of the SEC traces (differential refractive index (DRI) detector, THF, 1 mL min^–**1**^) of the “Staudinger” SCNPs after Me_3_SiOH triggered unfolding, **3′**, the “Staudinger” SCNPs before unfolding, **3**, and the initial functionalized precursor polymer, **2**. (C) IR spectra of **3** and **3′**, showing the latter the complete disappearance of the N=P stretching vibration band centered at 1175 cm^–**1**^ and new bands that can be assigned to vibrations of −CH_3_ groups. (D) ^**19**^F NMR spectra of **3** and **3′**, showing the latter the complete disappearance of the bands coming from −F atoms at *orto*- and *meta*-position with respect to azaylide groups. (E) ^**1**^H NMR spectra of Me_3_SiOH and **3′** showing the region in which protons from −CH_3_ groups bonded to the silicon atom appear. (F) DOSY-NMR experiment confirming that the −CH_3_ groups (denoted as **a′**)—having the same diffusion coefficient (*D*) as main chain −CH_2_– and −CH– protons (denoted as **b**)—belong to the polymer structure of **3′**. Signals with a diffusion coefficient faster than that of **3′** correspond to residual solvent impurities.

To investigate how robust the cross-linking N=P bonds of **3** are against oxidation, hydrolysis, and aza-Wittig reactions, we carried out several experiments. First, TGA results in an air atmosphere revealed that **3** is thermally stable up to 150 °C (see Experimental Section). Next, we investigated the stability of **3** in solution (5 mg mL^–1^) under different conditions and reagents through ^31^P NMR spectroscopy. As illustrated in Figure 3A, no changes in the peak position corresponding to the N=P cross-linking points were observed when **3** was heated at 120 °C for 3 days in dimethylformamide (DMF). Moreover, **3** in THF solution was found to be stable in the presence of an excess of either trifluoroacetic acid (CF_3_COOH) or carbon disulfide (CS_2_) for 3 days at r.t. All of these results supported the formation of robust “Staudinger” SCNPs containing stable azaylide moieties.

**Scheme 3:**
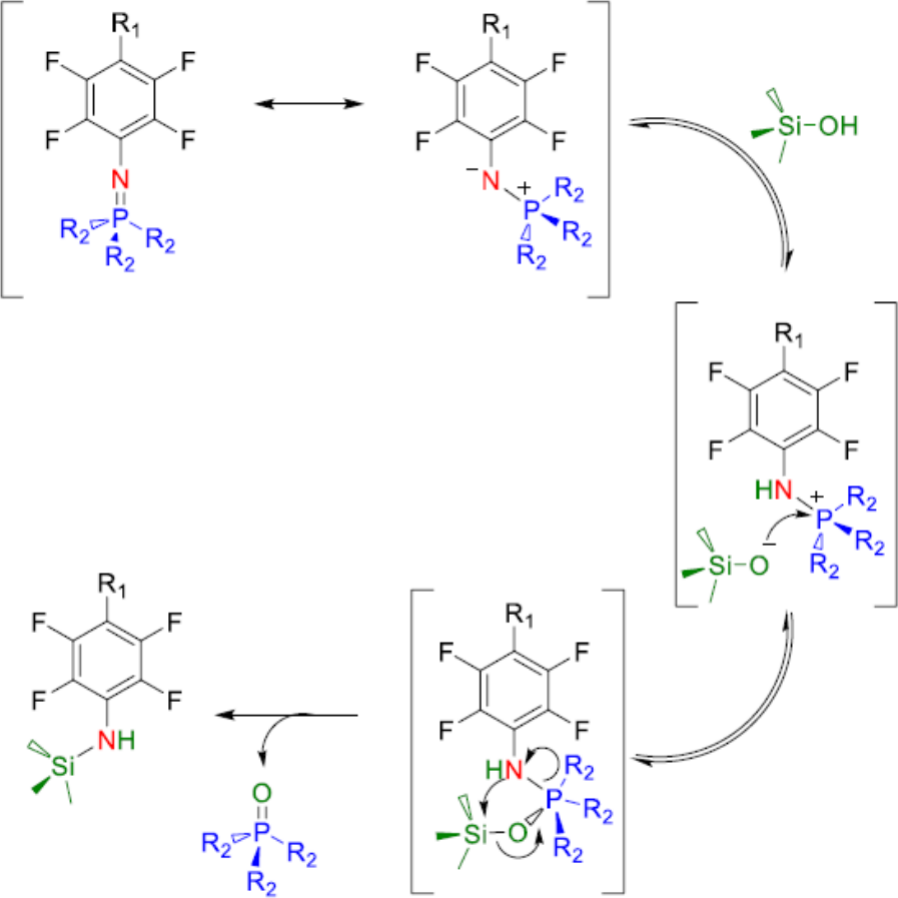
Tentative Mechanism of the Me_3_SiOH-Triggered Stable Azaylide Cleavage Reaction

At this stage, we realize that to break the robust N=P bonds in **3** and hence to promote the unfolding of the SCNPs, we should select a reagent presenting high affinity toward both the iminophosphorane linkage and −C_6_F_4_– ring while also exhibiting a nucleophilic character against the former. In this sense, silicon-based compounds become an attractive option not only because of the known affinity between Si and F but also because of their ability to be activated by phosphazene structures (iminophosphoranes).^56^ Thus, we hypothesized that trimethylsilanol (Me_3_SiOH) containing the “electropositive” silicon atom and having weak acidity (p*K*_a_ = 11) could be a good candidate.^57^ To our delight, when we treated a THF solution of **3** with an excess of Me_3_SiOH for 3 days at r.t. we observed the complete disappearance of the peak corresponding to the N=P cross-linking points in the ^31^P NMR spectrum of the resulting material (**3′**) (Figure 3A). Additionally, SEC results showed a shift toward shorter retention time and, hence, larger *R*_h_ (Figure 3B). In fact, the SEC retention times at the peak maximum of **3′** and the functionalized precursor **2** were found to be nearly identical (**3′**: *M*_w_ (MALLS) = 51.9 kDa, *Đ* = 1.1). Additional characterization of **3′** was carried out by combining a variety of techniques including IR and (^1^H, ^19^F) NMR spectroscopy. The IR spectrum of **3′** showed the complete disappearance of the N=P stretching vibration at 1175 cm^–1^ and the appearance of new bands that can be assigned to vibrations of the −CH_3_ groups (see Figure 3C). On one hand, the ^19^F NMR spectrum of **3′** confirmed the complete disappearance of the two peaks coming from −F atoms at *orto*- (δ = −155.2 ppm) and *meta*- (δ = −147.1 ppm) position with respect to azaylide groups (Figure 3D). On the other hand, the ^1^H NMR spectrum of **3′** showed a new band centered at δ = 0.11 ppm that can be assigned to protons of −CH_3_ groups, whereas in neat Me_3_SiOH the band of the protons from −CH_3_ groups was located at δ = 0.03 ppm (Figure 3E). Moreover, a DOSY-NMR experiment^58^ confirmed the −CH_3_ groups—having the same diffusion coefficient (*D*) as main chain −CH_2_– and −CH– protons—belong to the polymer structure of **3′** (see Figure 3F). Data from additional experiments are provided in the SI. Consequently, all the above results support that trimethylsilanol is a breakthrough, unique, and selective reagent for the efficient on-demand unfolding of the (otherwise) highly stable “Staudinger” SCNPs. A tentative mechanism of the trimethylsilanol-triggered stable azaylide cleavage reaction is provided in Scheme 3.

## Experimental Section

### Materials

Styrene (S) (≥99%), sodium azide (NaN_3_) (≥99%), *N,N*-dimethylformamide (DMF) (≥99.9%), cyanomethyl dodecyl carbonotrithioate (CMDTC) (98%), *n*-hexane (Hex) (≥97%), carbon disulfide (CS_2_) (≥99.9%), and trifluoroacetic acid (TFA) (≥98%) were purchased from Merk (Sigma-Aldrich). 1,3-Bis(diphenylphosphino)propane (DPPP) (>98%) and 1,4-bis(diphenylphosphino)butane (DPPB) (>98%) were supplied by TCI. Tetrahydrofuran (THF) (GPC grade) was purchased from Scharlau. 2,2′-Azobis(2-methylpropionitrile) (AIBN) (≥97%) was purchased from Fisher Scientific. 2,3,4,5,6-Pentafluorostyrene (PFS) (98%) and trimethylsilanol (Me_3_SiOH) (96.85%) were purchased from BLDpharm. Deuterated chloroform (CDCl_3_) (99.50% D, 0.03% TMS v/v) and deuterated tetrahydrofuran (THF-d_8_) (99.50% D, water <0.05%) were purchased from Eurisotop. Unless otherwise specified, all reagents were used as received without further purification. To remove the inhibitor, S and PFS were purified by passing through a column packed with activated basic alumina.

### Techniques

Elemental analysis (EA) measurements were performed in a Euro EA3000 elemental analyzer (CHNS). ^1^H, ^13^C, ^19^F, and ^31^P nuclear magnetic resonance (NMR) spectra were recorded at r.t. on a Bruker Ascend spectrometer operating at 400 MHz. CDCl_3_ was used as a solvent for the characterization of **1**, **2**, and **3**. THF-d_8_ was used as a solvent after the unfolding experiments. DOSY experiments were recorded at room temperature on a two-bay Bruker Avance spectrometer operating at 500 MHz, using THF-d_8_ as solvent. Fourier transform infrared (FTIR) spectra were recorded at room temperature on a JASCO 3600 FTIR spectrometer. Size exclusion chromatography (SEC) measurements were performed at 30 °C on an Agilent 1200 system equipped with PLgel 5 μm Guard and PLgel 5 μm MIXED-C columns, and triple detection: a differential refractive index (DRI) detector (Optilab Rex, Wyatt), a multiangle laser light scattering (MALLS) detector (MiniDawn Treos, Wyatt), and a viscosimetric (VIS) detector (ViscoStar-II, Wyatt). Data analysis was performed with ASTRA Software (version 6.1) from Wyatt. THF was used as the eluent at a flow rate of 1 mL min^–1^. A value of d*n*/d*c* = 0.100 mL g^–1^ was used for **1**, **2**, **3**, and **3′**. Dynamic light scattering (DLS) measurements were carried out at room temperature on a Malvern Zetasizer Nano ZS apparatus. Small-angle X-ray scattering (SAXS) experiments were conducted on the Rigaku 3-pinhole PSAXS-L equipment of the Materials Physics Center operating at 45 kV and 0.88 mA. The MicroMax-002+ X-ray Generator System is composed of a microfocus sealed tube source module and an integrated X-ray generator unit which produces CuK_α_ transition photons of wavelength λ = 1.54 Å. The flight path and the sample chamber in this equipment are under vacuum. The scattered X-rays are detected on a two-dimensional multiwire X-ray detector (Gabriel design, 2D-200X). This gas-filled proportional type detector offers a 200 mm diameter active area with ca. 200 μm resolution. After azimuthal integration, the scattered intensities were obtained as a function of momentum transfer *Q* = 4πλ^–1^ sin θ, where θ is half of the scattering angle. Reciprocal space calibration was done using silver behenate as standard. The sample-to-detector distance was 2 m, covering a *Q* range between 0.008 and 0.20 Å^–1^. The measurements were performed at rt on THF solutions in capillaries of 2 mm thickness. The concentration was 1 mg mL^–1^ in order to avoid interference effects between different macromolecules. The data were carefully corrected for background scattering (due to capillary and solvent) and measured for each sample on the same capillary for the same time. The generalized Gaussian coil function^59^ was employed for a precise determination of the values of the radius of gyration, *R*_g_, and scaling exponent, ν. Thermal gravimetric analysis (TGA) measurements were performed in a Q500-TA Instruments apparatus at a heating rate of 10 °C min^–1^ under air atmosphere from r.t. to 700 °C. Differential scanning calorimetry (DSC) measurements were carried out on ca. 5 mg specimens using a Q2000 TA Instruments apparatus in modulated mode. A helium flow rate of 25 mL min^–1^ was used throughout.

### Synthetic Procedures

#### Precursor Synthesis (**1**)

Precursor **1** was synthesized by RAFT polymerization in bulk. In a typical procedure, S (1 mL, 8.7 mmol), PFS (0.5 mL, 3.7 mmol), AIBN (1.4 mg) as a radical initiator, and CMDTC (2.6 mg) as a RAFT agent were added to a 50 mL round-bottom flask. After passing argon for 15 min, the reaction mixture was stirred at 65 °C for 15 h. **1** was obtained by precipitation in cold water, isolated by filtration, and finally dried in a vacuum oven at 35 °C overnight. **1**: yield = 61%; PFS content (EA) = 32 mol %; *M*_w_ (MALLS detector) = 42.6 kDa; *Đ* (SEC) = 1.08.

#### Precursor Functionalization (**2**)

**1** (0.5 g, 1.2 mmol of para-F groups) and NaN_3_ (0.17 g, 2.6 mmol, 2.2 equiv) were dissolved in DMF (25 mL) in a 100 mL round-bottom flask, and the resulting mixture was stirred at 80 °C for 24 h. **2** was recovered by precipitation in cold water and then filtered and dried at 35 °C under vacuum overnight. **2**: yield = 88%; azide content (^19^F NMR) = 10.5 mol %; *M*_w_ (MALLS) = 50.9 kDa; *Đ* (SEC) = 1.13.

#### “Staudinger”SCNPs (**3**)

**2** (100 mg, 0.08 mmol of para-N_3_ groups) was dissolved in THF (25 mL) in a flask that was sealed and degassed. In a second flask that was also sealed, degassed, and protected from light with aluminum foil, DPPP (20 mg, 0.05 mmol, 0.59 equiv) was dissolved in 175 mL of THF. The solution containing **2** was injected into the DPPP solution (both at r.t.) using an infusion pump at an addition rate of 30 mL h^–1^. After 24 h, the “Staudinger” SCNPs, **3**, were precipitated in cold hexane, filtered, and dried at 35 °C under a vacuum overnight. **3**: yield = 94.4%; *M*_w_ (MALLS) = 61.4 kDa; *Đ* (SEC) = 1.04.

#### Unfolded “Staudinger” SCNPs (**3′**)

In several 10 mL vials, **3** (10 mg, 0.008 mmol of N=P bonds) was dissolved in 2 mL of solvent (DMF or THF). Several experiments were carried out:

A closed 10 mL vial containing **3** in DMF was heated to 120 °C for 3 days. The resulting material after isolation was analyzed by ^31^P NMR spectroscopy showing **3** was stable under these conditions (unfolding did not take place).
A closed 10 mL vial containing **3** in DMF was exposed to microwave irradiation for 1 h (CEM Discover SP System, 25 W, 120 °C). **3** was found to be stable under these conditions.
A closed 10 mL vial containing **3** in THF and an excess of CF_3_COOH (2 mL, 18.0 mmol) was stirred at rt for 3 days. **3** was found to be stable under these conditions.
A closed 10 mL vial containing **3** in THF and an excess of CS_2_ (2 mL, 33.2 mmol) was stirred at rt for 3 days. **3** was found to be stable under these conditions.
A closed 10 mL vial containing **3** in THF and an excess of Me_3_SiOH (2 mL, 26.1 mmol) was stirred at rt for 3 days. The resulting material after isolation (**3′**) was analyzed by ^31^P NMR spectroscopy, showing the complete disappearance of the peak corresponding to cross-linking N=P bonds.

## Conclusions

In summary, Me_3_SiOH arises as a rare, exceptional, and valuable reagent for the cleavage of stable azaylides prepared by a nonhydrolysis Staudinger reaction, as revealed by the efficient unfolding of “Staudinger” SCNPs that are thermally stable up to 150 °C and their cross-linking N=P bonds are robust against strong acids like CF_3_COOH (p*K*_a_ = 0.23) as well as standard reagents for azaylides like CS_2_. This finding opens new possibilities for the disassembly of a variety of stable azaylide compounds prepared through a nonhydrolysis Staudinger reaction, such as bioconjugated molecules, fluorescent probes, or amphiphilic aggregates. Moreover, the Me_3_SiOH-triggered stable azaylide cleavage reaction provides a catalyst-free route to *N*-silylamines as valuable substrates in a number of different catalytic transformations (e.g., hydroamination of alkynes, hydroaminoalkylation of alkenes). Alternatively, the facile hydrolytic cleavage of *N*-silylamines upon reaction workup leads to the amine product of the classical Staudinger reaction. We are currently investigating the scope of the Me_3_SiOH-triggered stable azaylide cleavage reaction for other systems, including nonpolymeric substrates, and optimizing the reaction conditions (temperature, solvents, reaction time). The main results will be reported in due time.

## Supporting Information Available

Elemental analysis data; theoretical elemental composition; comparison of the DSC and TGA traces of **1**, **2**, and **3**; NMR spectra; DLS size distributions; and comparison of the SEC traces of “Staudinger” SCNPs (PDF)
