# Supplementary material for: Trimethylsilanol Cleaves Stable Azaylides As Revealed by Unfolding of Robust “Staudinger” Single-Chain Nanoparticles
Source: ACS Polym Au. 2024 Jan 9;4(2):140–8. doi: 10.1021/acspolymersau.3c00046 (PMC11010256; doi:10.1021/acspolymersau.3c00046)
Supplement: Supplementary file 1 — lg3c00046_si_001.pdf [file lg3c00046_si_001.pdf]

Supporting Information

Trimethylsilanol Cleaves Stable Azaylides as Revealed by Unfolding of Robust "Staudinger" Single-Chain Nanoparticles

Agustín Blázquez-Martín,[1] Sebastián Bonardd,[1,2] Ester Verde-Sesto,[1,3] Arantxa Arbe[1] and José A. Pomposo*,[1,2,3]

[1]Centro de Física de Materiales (CSIC - UPV/EHU) - Materials Physics Center MPC, Pº Manuel Lardizabal 5, E-20018 Donostia, Spain

[2]Departamento de Polímeros y Materiales Avanzados: Física, Química y Tecnología. University of the Basque Country (UPV/EHU), Pº Manuel Lardizabal 3, E-20800 Donostia, Spain

[3]IKERBASQUE – Basque Foundation for Science, Plaza Euskadi 5, E-48009 Bilbao, Spain

*Email: josetxo.pomposo@ehu.eus

1. Supplementary Tables

Table S1. Elemental analysis (EA) data.

**Sample**	**$C_{exp.}$ (wt. %)**	**$H_{exp.}$ (wt. %)**	**$N_{exp.}$ (wt. %)**
**1**	71.49	5.24	< 1.6
**2**	69.01	4.98	3.27
**3**	70.83	7.79	< 1.6

Table S2. Theoretical elemental composition.

**Sample**	**$C_{theor.}$ (wt. %)**	**$H_{theor.}$ (wt. %)**	**$N_{theor.}$ (wt. %)**
**1**	72.29[a]	4.82[a]	-
**2**	70.94[b]	4.73[b]	3.41[b]
**3**	73.47[c]	5.05[c]	0.99[c]

[a] According to x = 0.68 and y = 0.32 (Scheme 2, main text).
[b] According to x = 0.68, y-z = 0.21 and z = 0.11 (Scheme 2, main text).
[c] By assuming a degree of azaylide intrachain formation of 100%.

2. Supplementary Figures

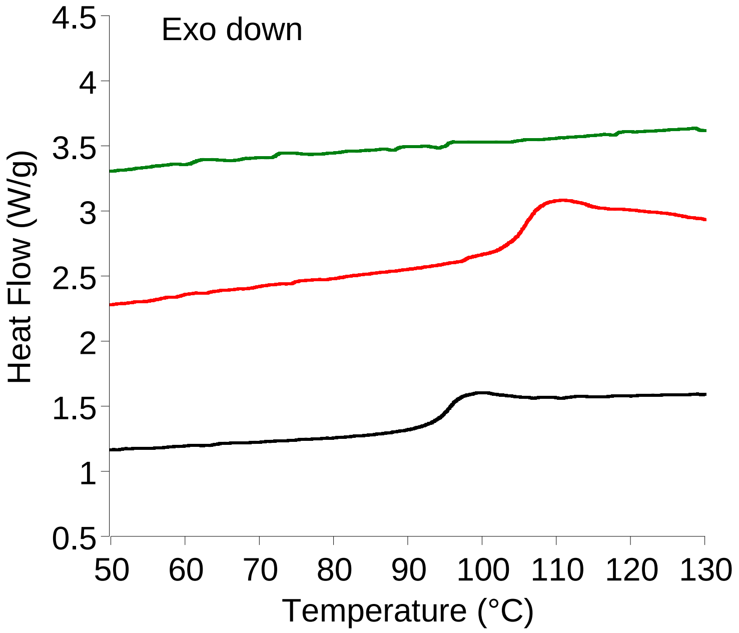

Figure S1. Comparison of the DSC traces of **1** (black color), **2** (red color) and **3** (green color).

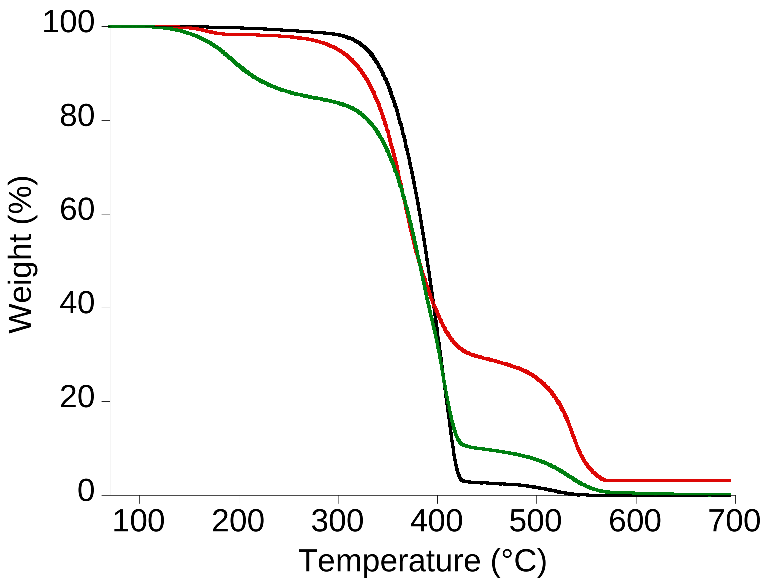

Figure S2. Comparison of the TGA traces of **1** (black color), **2** (red color) and **3** (green color).

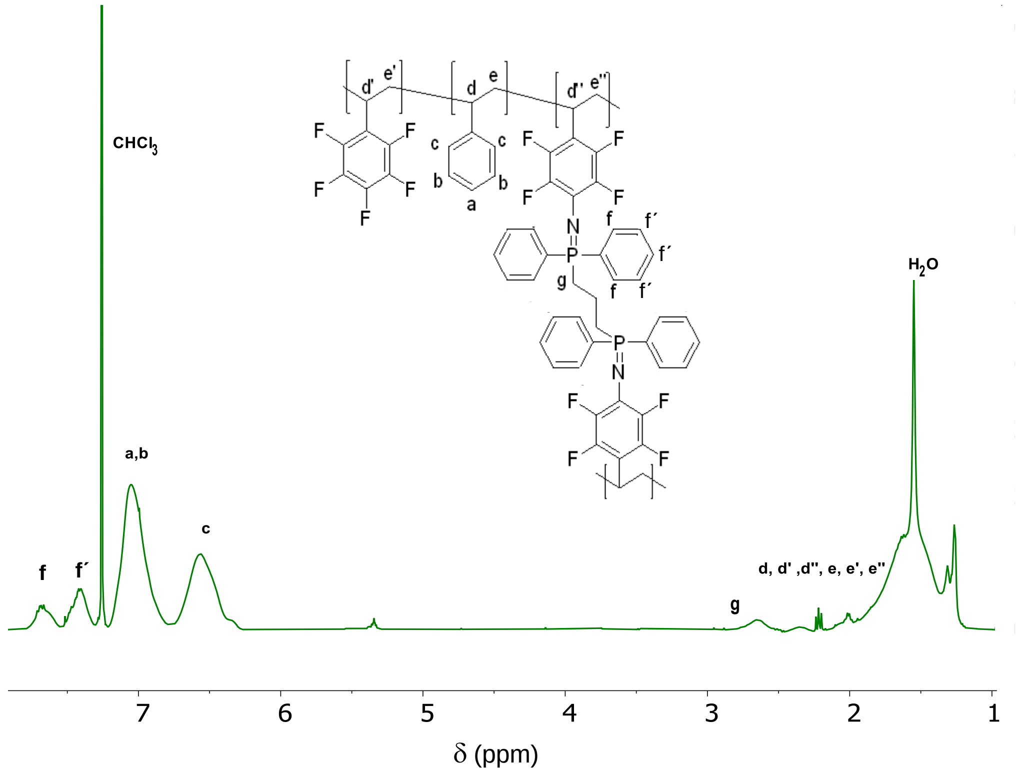

Figure S3. ^{1}H NMR spectrum of **3**.

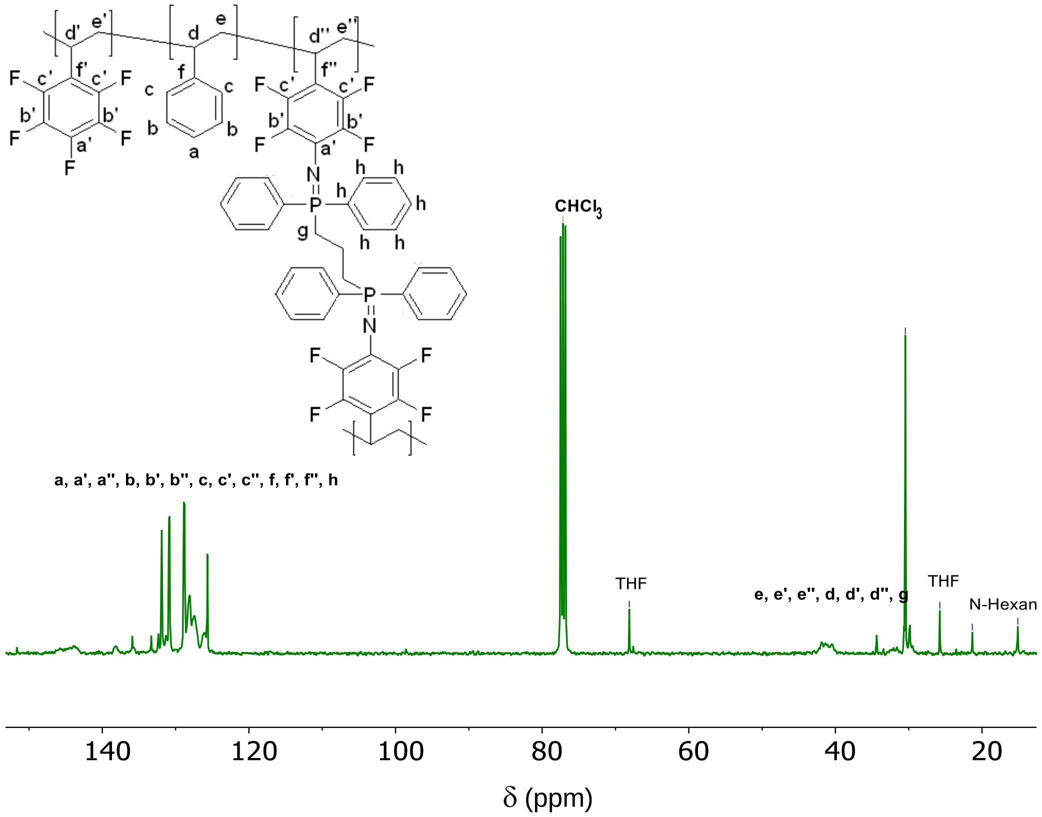

Figure S4. ^{13}C NMR spectrum of **3**.

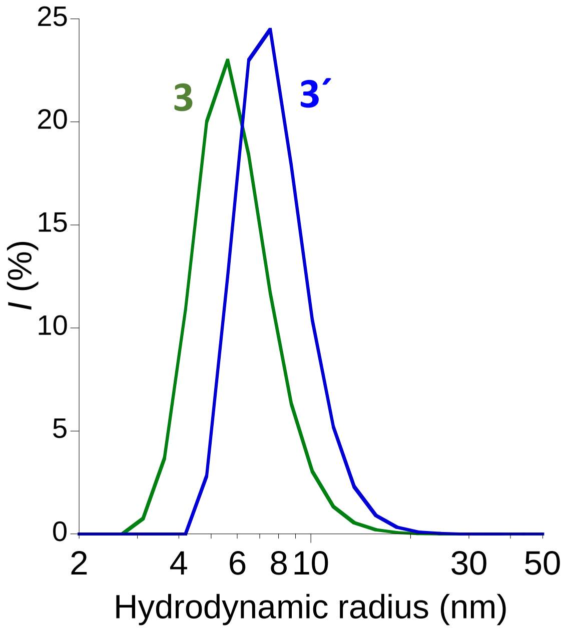

Figure S5. DLS size distributions of **3** and **3´** showing the successful Me_3SiOH triggered unfolding of the “Staudinger” SCNPs **3**.

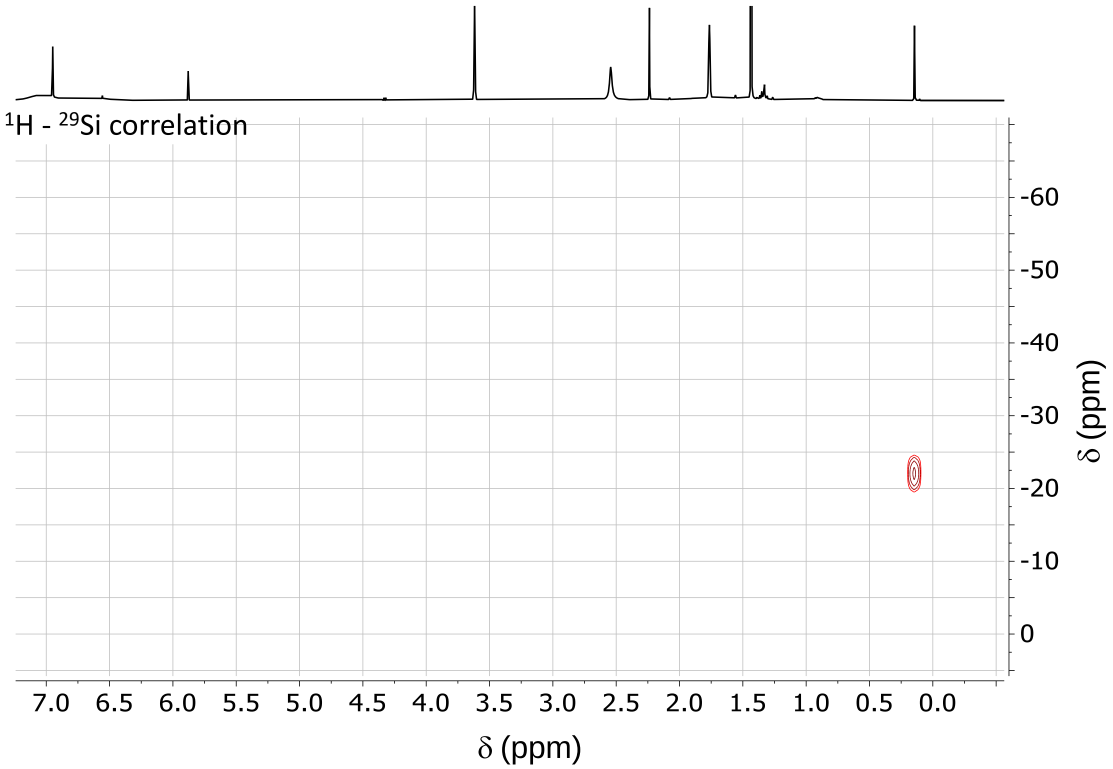

Figure S6. ^{1}H - ^{29}Si correlation NMR experiment showing that the $–CH_3$ groups of **3´** are attached to Si atoms.

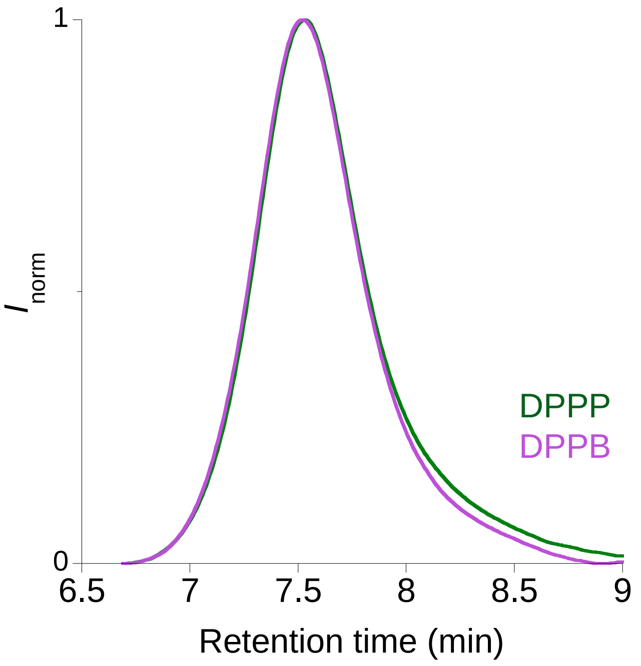

Figure S7. Comparison of the SEC traces of “Staudinger” SCNPs synthesized with 1,3-bis(diphenylphosphino)propane (DPPP) or 1,4-bis(diphenylphosphino)butane (DPPB) as intrachain cross-linker.
